# Performance Analysis of Cognitive Relay-Assisted Ambient Backscatter with MRC over Nakagami-*m* Fading Channels

**DOI:** 10.3390/s20123447

**Published:** 2020-06-18

**Authors:** Dinh-Thuan Do, Thanh-Luan Nguyen, Byung Moo Lee

**Affiliations:** 1 Wireless Communications Research Group, Faculty of Electrical & Electronics Engineering, Ton Duc Thang University, Ho Chi Minh City 700000, Vietnam; 2 Faculty of Electronics Technology, Industrial University of Ho Chi Minh City (IUH), Ho Chi Minh City 700000, Vietnam; nguyenthanhluan@iuh.edu.vn; 3 School of Intelligent Mechatronics Engineering, Sejong University, Seoul 05006, Korea

**Keywords:** ambient backscatter, cognitive radio, outage probability

## Abstract

This study presents ambient backscatter communication (AmBC) network as a concept of "modulation in the air" that has drawn growing interest by both academia and industry recently. In particular, we investigate and analyze an AmBC system relying on cognitive radio, where the primary destination is equipped with multiple antennas and maximum ratio combining (MRC). A wireless powered relay is necessary to serve both primary and secondary destinations. Benefiting from the surrounding radio frequency (RF) source, the relay can support the backscattering signal. To facilitate the performance analysis of received nodes, this study presents exact closed-form expressions of the outage probability. For comparison, the outage and throughput performance of these nodes are considered in numerical simulation. Taking advantage of the AmBC technology, the impact of the backscatter ratio on system performance is carefully studied considering various other parameters. Simulation results demonstrate the exactness of the derived outage probabilities and show that the optimal throughput performance can be achieved at specific parameters.

## 1. Introduction

As an emerging technique in terms of spectrum- and power-efficient systems, ambient backscatter communication (AmBC) can be recommended to implement green Internet-of-Things (IoT) [1,2,3]. In principle, AmBC devices can harvest power from surrounding popular radio frequency (RF) sources such as wireless fidelity (Wi-Fi) access points (AP), digital television (DTV) transmitters, cellular base stations (BS). AmBC devices employ harvested energy to power its circuit and modulate its own signal over the RF source signal [1]. The backscatter device (BD), ambient radio-frequency (RF) source, and reader are three components in AmBC. Concerning two features, such as reflecting the signals from the ambient RF source (legacy transmitter) and using common RF components, the BD sends a signal to the reader through adjusting its load impedance [1]. Therefore, research on AmBC networks can be seen in both academia and industry for implementing future IoT [2,3,4,5,6,7,8,9,10]. To maximize the ergodic rate of the BD, the authors in [4] studied the optimal BD reflection coefficient, and the optimal RF source transmitted power. By examining capacity, the authors in [5] explored AmBC over the legacy orthogonal frequency division multiplexing (OFDM) signal. The cooperative AmBC network is considered in [6], in which the reader recovers information not only from the AmBC device but also from the RF source. They evaluate the system model’s performance for the AmBC system over ambient orthogonal frequency division multiplexing carriers for frequency-selective fading channels. The authors in [10] investigated outage performance of AmBC and provided an emerging green communication framework that allows passive devices to communicate with each other through exploiting the environmental radio frequency signals.

In other emerging networking paradigms, wireless powered communication (WPC) networks have been recently proposed [11,12,13,14,15]. In WPC systems, wireless power transfer technology is manipulated to replenish the energy-constrained devices remotely. Unlike battery-powered networks, the WPC networks improve network lifetime with a significant reduction of operational cost without requiring any manual replacement or recharging of batteries [11,12,13]. Ambient RF energy harvesting (EH) provides two advantages, such as reliability and more flexible solution in powering devices. EH technique outperforms other conventional EH techniques such as solar, wind, and thermoelectric energy, which mainly depend on the surrounding environments. Besides reliability and flexibility, the ability of the RF signals to simultaneously carry both information and energy can be considered as another major advantage of the RF-EH scheme [15,16,17,18,19,20]. The simultaneous wireless information and power transfer (SWIPT) technique was introduced along with its performance in [17]. Two modes, including time-switching (TS) and power-splitting (PS), are studied to facilitate SWIPT in practical scenarios [18]. The TS scheme divides the time domain to switch between information processing and EH. Unlike TS, EH and information processing are proceeded in different power domains in the PS scheme. The optimal throughput in energy-aware cooperative networks can be achieved in the two-way relaying networks (TWRN) under the framework of simultaneous time and power energy harvesting protocol, namely time power switching (TPS) based relaying [20]. In particular, the impact of imperfect hardware at the relay and the destination node, the system performance was analyzed in outage behavior and throughput performance [20].

In order to realize high energy and spectrum efficiency, it is possible to integrate AmBC into RF-powered radio networks [21,22,23,24,25,26,27,28,29,30,31]. The authors in [22] presented hybrid backscatter communication for wireless-powered communication networks to intensify transmission coverage and provide uniform rate distribution in the heterogeneous network (HetNet) manner. They derived formulas of a throughput maximization problem relying on the user location. A novel opportunistic ambient backscatter communication AmBC framework is further studied to implement radio frequency (RF)-powered cognitive radio (CR) networks [28]. They derived analytic expressions in terms of the average energy consumption, the average throughput, and energy efficiency. In [29], the AmBack-assisted CR network is proposed with the capability of energy harvesting under a scenario that the primary user (PU) was occupying the spectrum. In particular, the data transmission of the cognitive user (CU) can be employed, relying on the energy harvested from the PU. The proposed CR does not require perfect symbol synchronization between the IoT and the primary transmissions. By jointly optimizing the time-sharing and power allocation coefficients, the authors in [30] presented the maximal data rate of the IoT transmission subject to the minimum rate requirement of the primary system for a cognitive backscatter network. The AmBC-enabled cognitive relay network is explored by enabling decode-and-forward (DF) relay and wireless energy-harvesting capabilities [31]. In this system, the relay node can be operated with two different modes. The relay can further concurrently decode/harvest and backscatter the received signals.

Motivated by previous analysis, this paper addresses how to improve the selection of primary destination. This open problem has not been well studied in [28,29,30,31]. The main findings of the present paper are summarized as follows.

- The AmBC and SWIPT improve performance of distance user in cognitive radio network, and this paper studies ability of the relay node to concurrently perform both SWIPT and AmBC operations.
- Since the proposed network benefit by multiple antennas designed at the primary destination, a maximal ratio combining (MRC) mechanism is adopted at non-AmBC destination. For the evaluation of system performance, the impact of AmBC on two destinations is studied in two main metrics, such as outage probability and achievable throughput.
- For both the primary and secondary communications, we derive the closed-form formula of both the throughput and the outage probability. We verify theoretical computation via simulation results and also evaluate the effect of different system parameters on the system performance.

Table 1 provides all of the abbreviations and acronyms used in our paper.

The rest of this paper is organized as follows. Section 2 presents the considered system model and a detailed computations of received signals related to SWIPT and AmB operations. Section 3 derives analytical expressions for the outage probability and the achievable throughput. The performance analysis using numerical simulation and the conclusion of this paper are presented in Section 4 and Section 5, respectively.

## 2. System Model

### 2.1. System Model

**Figure 1:**
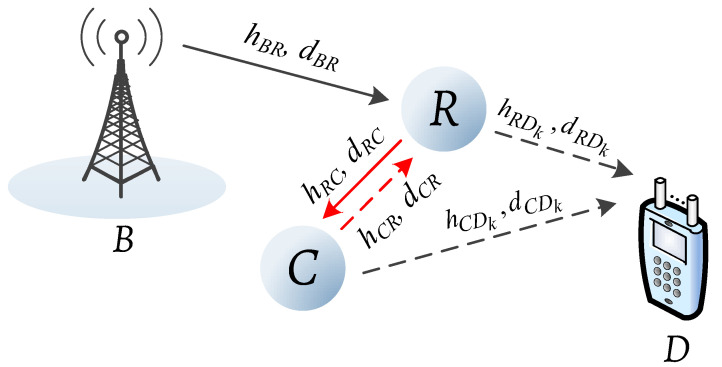
System model of the proposed AmBC network which comprises a base station *B*, a relay *R*, an AmB transceiver node *C* and a *K*-antenna destination *D*.

In this paper, a cognitive radio-based AmBC network demonstrated in Figure 1 is considered in term of system performance. The network consists of a primary base station *B*, an energy-constrained relay *R*, a destination *D*, and a secondary AmBack transceiver *C*. The relay holds the ability to simultaneously execute SWIPT and backscatter transmission via the two specialized modules at this transceiver. Particularly, the SWIPT module is equipped with decode-and-forward (DF) relaying mode to forward the primary information from node *B* to node *D*, and the AmBack module is employed for the secondary communication with node *C*. It is recalled that the relay has limited power, and a PS-based relaying protocol is adopted to support its operations. In addition, due to potential interference from node *C*, the destination is facilitated with *K* antennas and the capability to perform MRC to further enhance its own performance.

In the considered hybrid SWIPT-AmBC model, the channel coefficients of the links *B*-to-*R*, *R*-to-*C*, *C*-to-*R*, *R*-to-*D* and *C*-to-*D* are denoted by $h_{BR}$, $h_{RC}$, $h_{CR}$, $h_{RD_{k}}$ and $h_{CD_{k}}$ for k=1,2,…,K, respectively, and the corresponding distances are $l_{BR}$, $l_{RC}$, $l_{CR}$, $l_{RD_{k}}$ and $l_{CD_{k}}$, respectively.

Further, we assume a block-fading model where all channels between the nodes remain constant over any given time block and independently distributed among different ones. The channel gains between node *U* and node *V* for $UV\in \{BR,RC,CR,RD_{k},CD_{k}\}$ are modeled by Nakagami-*m* fading with integer shapes $m_{UV}$ and unit variances. It is worth noting that the probability density function (PDF) and the cumulative distribution function (CDF) of a squared Nakagami-*m* fading with integer shape $m_{UV}$ and variance $\lambda_{UV}$, denoted by $X_{UV}$, are given respectively by

(1) $$\begin{array}{c} f_{X_{UV}}\left(x\right)=\frac{1}{\left(m_{UV}-1\right)!}{\left(\frac{m_{UV}}{\lambda_{UV}}\right)}^{m_{UV}}x^{m_{UV}-1}\exp\left(-\frac{m_{UV}}{\lambda_{UV}}x\right),\;\phi >0, \end{array}$$

(2) $$\begin{array}{c} F_{X_{UV}}\left(\phi \right)=1-\exp\left(-\frac{m_{UV}}{\lambda_{UV}}x\right)\sum_{m=0}^{m_{UV}-1}\frac{x^{m}}{m!}{\left(\frac{m_{UV}}{\lambda_{UV}}\right)}^{m},\;\phi >0. \end{array}$$

### 2.2. Energy Harvesting, Information Decoding, and Ambient Backscatter Operations

**Figure 2:**
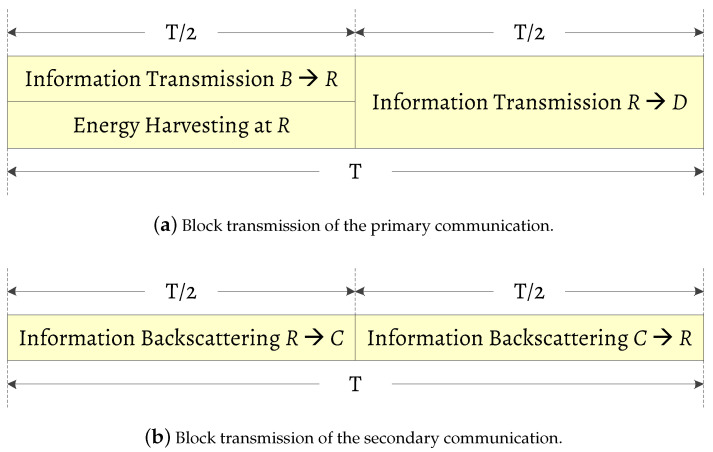
Transmission model of the proposed network.

The transmission of the information signal from the node *B* to the destination *D* is taken over a duration of *T* block time, as depicted in Figure 2. During the first half block time T/2, the source node *S* transmits its information signal with power $P_{B}$ to the relay, the relay then adopts PS scheme to split a portion of the received power ($\alpha P_{B}$) for EH and the remaining power ($\left(1-\alpha \right)P_{B}$) for information transmission (IT). Herein, 0≤α≤1 is the power splitting ratio, which can be tuned at the relay. Simultaneously, the relay also backscatters its secondary information signal to the AmBC transceiver *C*. During the second half block time, the relay forwards the source signal to the node *D* while the node *C* backscatters its secondary signal to the relay. Accordingly, the received signal at the information receiver in the relay can be formulated by

(3) $$\begin{array}{c} y_{SR}=\sqrt{\left(1-\alpha \right)P_{B}}\frac{h_{BR}}{\sqrt{l_{BR}^{\epsilon }}}x_{B}+n_{R}, \end{array}$$

where $x_{B}$ and $n_{R}$ are the normalized source signal, i.e., $E[|x_{B}{|}^{2}]=1$, and the zero mean additive Gaussian noise (AWGN) with variance $\sigma^{2}$.

Subsequently, the received signal-to-noise ratio (SNR) at the relay is expressed as

(4) $$\begin{array}{c} \gamma_{BR}=\left(1-\alpha \right)\frac{{\bar{P}}_{B}}{l_{BR}^{\epsilon }}{\left|h_{BR}\right|}^{2}, \end{array}$$

in which ${\bar{P}}_{B}=P_{B}/\sigma^{2}$ specifies the average transmit SNR. As mentioned, the relay uses portion of the received power for energy harvesting and thus the harvested energy via PS protocol is given by

(5) $$\begin{array}{c} \varepsilon_{PSR}=\alpha \eta \frac{P_{B}}{l_{BR}^{\epsilon }}{\left|h_{BR}\right|}^{2}\frac{T}{2}, \end{array}$$

in which 0≤η≤1 is the efficiency factor and its value depends on the designed EH circuitry. Note that the amount of energy harvested from the AWGN power is insignificant and thus can be neglected [19]. Assuming that the relay consumes all of the harvested energy for forwarding the source signal to node *D*, the transmit power (the harvested power) of the relay is obtained as

(6) $$\begin{array}{c} P_{R}\left(\alpha \right)=\frac{\varepsilon_{PSR}}{T/2}=\alpha \eta \frac{P_{B}}{l_{BR}^{\epsilon }}{\left|h_{BR}\right|}^{2}, \end{array}$$

In the network, the relay decodes $x_{B}$ correctly if the received instantaneous SNR $\gamma_{BR}$ exceeds the decoding threshold ${\bar{\gamma }}_{P}=2^{2{\bar{R}}_{P}}-1$ in which ${\bar{R}}_{P}$ (bits/s/Hz) denotes the data rate of the primary signal. Consequently, the value of the ratio α to ensure the decoding of $x_{B}$ is obtained by solving $\gamma_{BR}={\bar{\gamma }}_{P}$, thus

(7) $$\begin{array}{c} \alpha^{*}=1-{\bar{\gamma }}_{P}\frac{l_{BR}^{\epsilon }}{{\bar{P}}_{B}{\left|h_{BR}\right|}^{2}}. \end{array}$$

**Remark** **1.**  The value of the ratio α can be dynamically tuned at the relay R to satisfy the decoding condition of $x_{B}$ from the base station. Specifically, α is set to $\alpha^{*}$ expressed in the above equation. It should be pointed out that traditional EH-assisted systems adopt the PS scheme with fixed α [15,16,17,18,19,20]. Moreover, the value of α is independent of the surrounding environments, i.e., the channel from B to R. However, a dynamic value of α can be obtainable as the relay also requires the CSI of the B-R link to decode $x_{B}$, and thus can take advantage of this information to dynamically tune α and optimize the system performance.

In addition, if the relay tunes α beyond $\alpha^{*}$, less allocated power is reserved for information processing, which then results in such a failure in decoding of $x_{B}$. In addition, tuning $\alpha <\alpha^{*}$ results in lower transmit power during the second time block and could potentially harm the decoding process at node *D*, not to mention the extra power wasted for the decoding of $x_{B}$. Subsequently, the optimal transmit power of the relay is given by

(8) $$\begin{array}{c} P_{R}\left(\alpha^{*}\right)=\max\{0,\eta \left(\frac{{\bar{P}}_{B}}{l_{BR}^{\epsilon }}{\left|h_{BR}\right|}^{2}-{\bar{\gamma }}_{P}\right)\sigma^{2}\}. \end{array}$$

During the first time block, the relay *R* also backscatters its own signal to the AmBC node *C* via the AmBC module. Accordingly, the received SNR to decode the secondary signal can be formulated as [10]

(9) $$\begin{array}{c} \gamma_{RC}=\theta {\bar{P}}_{B}\frac{|h_{BR}{|}^{2}}{l_{BR}^{\epsilon }}\frac{|h_{RC}{|}^{2}}{l_{RC}^{\epsilon }}, \end{array}$$

in which 0≤θ≤1 denotes the backscattering ratio.

In the second half time block T/2, the relay *R* transmits the decoded signal to the destination *D* with the transmit power $P_{R}\left(\alpha \right)$. Simultaneously, the AmB node *C* also backscatters the received signal to the relay *R*.

It is worth pointing out that the relay operates in full-duplex mode. Particularly, the relay simultaneously receives the source’s signal and transmits (backscatters) its own signal to the AmBC receiver in the first half time block T/2. In addition, the relay also concurrently forwards the source’s signal to the destination and receives the backscattered signal from the node *C* in the second half time block T/2. In both time blocks, the secondary backscattered signal is modulated with the primary signals in the transmitter side, i.e., the relay node and the node *C*. Then, self-interference cancellation is performed at the receiver sides to retrieve the secondary signals.

At the node *D*, the weighted signal observed at the antennas is obtained by

(10) $$\begin{array}{c} y_{RD}^{MRC}=\sqrt{P_{R}\left(\alpha \right)}\frac{w_{MRC}^{H}h_{RD}}{\sqrt{l_{RD}^{\epsilon }}}{\hat{x}}_{B}+\sqrt{\theta P_{R}\left(\alpha \right)}\frac{h_{RC}}{\sqrt{l_{RC}^{\epsilon }}}\frac{w_{MRC}^{H}h_{CD}}{\sqrt{l_{CD}^{\epsilon }}}+w_{MRC}^{H}n_{D}, \end{array}$$

in which $h_{RD}={\left[h_{RD_{1}},h_{RD_{2}},\ldots ,h_{RD_{K}}\right]}^{T}$, $h_{CD}={\left[h_{CD_{1}},h_{CD_{2}},\ldots ,h_{CD_{K}}\right]}^{T}$, $w_{MRC}\in C^{K\times 1}$ specifies the antenna weights and $n_{D}$ is the AWGN vector with entries are complex normal RVs having zero means and variances $\sigma^{2}$. The weight vector aligned for MRC is $w_{MRC}=h_{RD}$, thus by following similar analysis in [32], the received signal-to-interference-plus-noise ratio (SINR) at node *D* in the presence of the co-channel interference from AmBC node *C* can be formulated by

(11) $$\begin{array}{cc} \gamma_{RD}^{MRC}= & \frac{P_{R}\left(\alpha \right)\frac{|h_{RD}^{H}h_{RD}|}{l_{RD}^{\epsilon }}}{P_{R}\left(\alpha \right)\theta \frac{|h_{RC}{|}^{2}}{l_{RC}^{\epsilon }}\frac{|h_{RD}^{H}h_{CD}{|}^{2}}{|h_{RD}^{H}h_{RD}|}\frac{l_{RD}^{\epsilon }}{l_{CD}^{\epsilon }}+\sigma^{2}} \end{array}$$

(12) $$\begin{array}{cc} = & \frac{\frac{P_{R}\left(\alpha \right)}{\sigma^{2}}X_{RD}}{\frac{P_{R}\left(\alpha \right)}{\sigma^{2}}X_{RC}X_{CD}+1}, \end{array}$$

where $X_{BR}={\left|h_{BR}\right|}^{2}l_{BR}^{-\epsilon }$, $X_{RD}=|h_{RD}^{H}h_{RD}|l_{RD}^{-\epsilon }$, $X_{RC}=\theta |h_{RC}{|}^{2}l_{RC}^{-\epsilon }$ and $X_{CD}=\frac{|h_{RD}^{H}h_{CD}{|}^{2}}{|h_{RD}^{H}h_{RD}|}l_{RD}^{\epsilon }l_{CD}^{-\epsilon }$. Note that $X_{CD}$ and $X_{RD}$ are independent RVs.

Similarly, the achievable SNR at the relay node from the AmBC node *C* can be given by

(13) $$\begin{array}{cc} \gamma_{CR}= & \frac{\theta |h_{CR}{|}^{2}{\left|h_{CR}\right|}^{2}P_{R}\left(\alpha \right)}{l_{BR}^{\epsilon }l_{RC}^{\epsilon }l_{CR}^{\epsilon }\sigma^{2}} \end{array}$$

(14) $$\begin{array}{cc} = & \theta X_{CR}^{2}\frac{P_{R}\left(\alpha \right)}{\sigma^{2}}, \end{array}$$

where $X_{CR}={\left|h_{CR}\right|}^{2}l_{CR}^{-\epsilon }$. It should be noted that $X_{CR}=X_{RC}/\theta$ due to channel reciprocal and block fading assumption.

## 3. System Performance Analysis

### 3.1. Preliminary Results

In this section, the probability density function (PDF) and the cumulative distribution function (CDF) of the random variables (RVs) those are essential for the analysis in the next section. We reiterate that for the random variable $X_{UV}$ with UV∈{BR,RC,CR}, the PDF and CDF are given by (1) and (2), respectively, in which $\lambda_{UV}=l_{UV}^{-\epsilon }$. Now, one can rewrite $|h_{RD}^{H}h_{RD}|l_{RD}^{-\epsilon }$ as $\sum_{k=1}^{K}{\left|h_{RD_{k}}\right|}^{2}l_{RD}^{-\epsilon }=X_{RD}$, which is the sum of *K* independent and identically distributed RVs, thus $X_{RD}$ is also a Gamma distributed RV. Specifically, the PDF and the CDF of $X_{RD}$ are expressed as

(15) $$\begin{array}{c} f_{X_{RD}}\left(x\right)=\frac{1}{\left(Km_{RD}-1\right)!}{\left(\frac{m_{RD}}{\lambda_{RD}}\right)}^{Km_{RD}}x^{Km_{UV}-1}\exp\left(-\frac{xm_{RD}}{\lambda_{RD}}\right),\;x>0, \end{array}$$

(16) $$\begin{array}{c} F_{X_{RD}}\left(x\right)=1-\exp\left(-\frac{m_{UV}}{\lambda_{RD}}x\right)\sum_{m=0}^{Km_{RD}-1}\frac{x^{m}}{m!}{\left(\frac{m_{RD}}{\lambda_{RD}}\right)}^{m},\;x>0. \end{array}$$

In addition, since $|h_{CD_{k}}{|}^{2}l_{CD}^{-\epsilon }$’s are independent and identical, the RV $X_{CD}$ can then be modelled by Gamma distribution with shape $m_{CD}$ and variance $\lambda_{CD}=l_{CD}^{-\epsilon }$ [33,34]. Hence, the PDF and CDF of $X_{CD}$ can be obtained via (1) and (2) with UV≡CD, respectively.

It can be noticed that the denominator of (12) contains the product of two independent Gamma RVs $X_{RC}$ and $X_{CD}$. In general, if $Z=X_{1}X_{2}\ldots X_{N}$ is the product of *N* independent and not necessarily identically distributed Gamma RVs where each $X_{n}$ (n=1,2,…,N) has real shape $m_{n}$ and variance $\mu_{n}$, the PDF of *Z* can be obtained by

(17) $$\begin{array}{c} f_{Z}\left(z\right)=\left(\prod_{n=1}^{N}\frac{1}{\Gamma \left(m_{n}\right)}\frac{m_{n}}{\mu_{n}}\right)G_{0,N}^{N,0}\left(\begin{array}{c} -\\ m_{1}-1,\ldots ,m_{N}-1 \end{array}|z\prod_{n=1}^{N}\frac{m_{n}}{\mu_{n}}\right), \end{array}$$

in which Γ(x) is the Gamma function and $G_{p,q}^{m,n}\left(\cdot \right)$ denotes the Meijer G-function.

**Proof.**  See Appendix A. □

Adopting the above equation with N=2, $\left(m_{1},\mu_{1}\right)=\left(m_{RC},\lambda_{RC}\right)$, $\left(m_{2},\mu_{2}\right)=\left(m_{CD},\lambda_{CD}\right)$ the pdf of $X_{RC}X_{CD}$ can be further derived by

(18) $$\begin{array}{cc} f_{X_{RC}X_{CD}}\left(x\right)= & \frac{1}{\left(m_{RC}-1\right)!\left(m_{CD}-1\right)!}\frac{m_{RC}}{\lambda_{RC}}\frac{m_{CD}}{\lambda_{CD}}G_{0,2}^{2,0}\left(\begin{array}{c} -\\ m_{RC}-1,m_{CD}-1 \end{array}|\frac{m_{RC}}{\lambda_{RC}}\frac{m_{CD}}{\lambda_{CD}}x\right) \end{array}$$

(19) $$\begin{array}{cc} = & \frac{1}{\left(m_{RC}-1\right)!\left(m_{CD}-1\right)!}\frac{A}{2}{\left(\frac{Ax}{4}\right)}^{\frac{m_{RC}+m_{CD}}{2}-1}K_{m_{RC}-m_{CD}}\left(\sqrt{Ax}\right),\;x>0, \end{array}$$

where the last equality is obtained by using [35] (Equation (9.34.3)) and $A=4m_{RC}m_{CD}/\lambda_{RC}\lambda_{CD}$.

### 3.2. Outage Probability Analysis

In this section, the proposed network’s performance is studied via the outage probability at the destination node *D*. Notably, the event that the node *D* cannot recover the primary signal $x_{B}$, which is determined by whether the SINR at the relay or the destination falls below the decoding threshold ${\bar{\gamma }}_{P}$ or not. In other words, the outage probability can be formulated by

(20) $$\begin{array}{cc} {\mathrm{OP}}_{D}^{out}= & \mathrm{Pr}\{\gamma_{BR}<{\bar{\gamma }}_{P}\;\mathrm{or}\;\gamma_{RD}^{MRC}<{\bar{\gamma }}_{P}\}. \end{array}$$

**Proposition** **1.**  The outage probability of the primary link, ${\mathrm{OP}}_{D}^{out}$, at $\alpha =\alpha^{*}$ can be expressed as

(21) $$\begin{array}{cc} {\mathrm{OP}}_{D}^{out}= & 1-\sum_{m=0}^{Km_{RD}-1}{\left(\frac{m_{RD}{\bar{\gamma }}_{P}}{\lambda_{RD}}\right)}^{m}\sum_{m_{1}+m_{2}=m}\frac{P_{1}\left(m_{1}\right)}{m_{1}!}\frac{P_{2}\left(m_{2}\right)}{m_{2}!}, \end{array}$$

*in which $P_{1}\left(m_{1}\right)$, $P_{2}\left(m_{2}\right)$ are given by*

$$\begin{array}{cc} P_{1}\left(m_{1}\right)= & \frac{\left(m_{RC}+m_{1}-1\right)!\left(m_{CD}+m_{1}-1\right)!}{\left(m_{RC}-1\right)!\left(m_{CD}-1\right)!}\frac{\sqrt{A}}{2}{\left(\frac{A}{4}\right)}^{\frac{m_{RC}+m_{CD}}{2}-1}\exp\left(\frac{A}{8}\frac{\lambda_{RD}}{m_{RD}{\bar{\gamma }}_{P}}\right) \end{array}$$

(22) ×(λRDmRDγ¯P)mRC+mCD2+m1−12W−mRC+mCD2−m1+12,mRC−mCD2(A4λRDmRDγ¯P),P2(m2)=2(mBR−1)!exp(−mBRγ¯PP¯BλBR)(1P¯BmBRηλBR)m2∑k=0mBR−1(mBR−1k)(mBRγ¯PP¯BλBR)k

(23) $$\begin{array}{cc} \; & \times {\left(\frac{{\bar{\gamma }}_{P}}{{\bar{P}}_{B}}\frac{m_{RD}}{\lambda_{RD}}\frac{m_{BR}}{\eta \lambda_{BR}}\right)}^{\frac{m_{BR}-k-m_{2}}{2}}K_{m_{BR}-k-m_{2}}\left(2\sqrt{\frac{{\bar{\gamma }}_{P}}{{\bar{P}}_{B}}\frac{m_{RD}}{\lambda_{RD}}\frac{m_{BR}}{\eta \lambda_{BR}}}\right), \end{array}$$

*respectively, where $W_{\mu ,v}\left(x\right)$ denotes the Whittaker-W function.*

**Proof.**  See Appendix B. □

Accordingly, the throughput of the primary link can be expressed as a function of the outage probability at node *D* as

(24) $$\begin{array}{c} C_{D}=\left(1-P_{D}^{out}\right){\bar{R}}_{P}\left(T/2\right)/T. \end{array}$$

In addition, the probability for the SNR at the AmBC node *C* to decode the secondary information falls below the decoding threshold ${\bar{\gamma }}_{S}$ is formulated by

(25) $$\begin{array}{c} {\mathrm{OP}}_{C}^{out}=\mathrm{Pr}\left\{\gamma_{RC}<{\bar{\gamma }}_{S}\right\}, \end{array}$$

in which ${\bar{\gamma }}_{S}=2^{2{\bar{R}}_{S}}-1$ with ${\bar{R}}_{S}$ (bits/s/Hz) being the transmission rate of the secondary signal. Accordingly, the above probability can be expressed in the analytical closed-form via the following proposition.

**Proposition** **2.**  *The probability for failed decoding, i.e., the outage probability, at the secondary AmB node C can be obtained using*

(26) $$\begin{array}{cc} {\mathrm{OP}}_{C}^{out}= & 1-\frac{1}{\left(m_{RC}-1\right)!}\sum_{m=0}^{m_{BR}-1}\frac{1}{m!}{\left(\frac{m_{BR}{\bar{\gamma }}_{S}}{\lambda_{BR}{\bar{P}}_{B}}\frac{m_{RC}}{\lambda_{RC}}\right)}^{m}\\ \; & \times 2{\left(\frac{m_{BR}{\bar{\gamma }}_{S}}{\lambda_{BR}{\bar{P}}_{B}}\frac{m_{RC}}{\lambda_{RC}}\right)}^{\frac{m_{RC}-m}{2}}K_{m_{RC}-m}\left(2\sqrt{\frac{m_{BR}{\bar{\gamma }}_{S}}{\lambda_{BR}{\bar{P}}_{B}}\frac{m_{RC}}{\lambda_{RC}}}\right). \end{array}$$

In the second time block, the node *C* backscatters its own signal to the relay *R* by exploiting the RF signal transmitted from the relay. Recalling that the relay forwards the decoded primary signal (${\hat{x}}_{B}$) to the destination if the ${\hat{x}}_{B}$ is successfully recovered during the first half block time T/2 otherwise remains silent. In the case of no transmission occurred, no backscattering operation is performed. Accordingly, the outage probability at the relay to decode the backscattered secondary signal is formulated by

(27) $$\begin{array}{cc} {\mathrm{OP}}_{R}^{out}= & 1-\mathrm{Pr}\{\gamma_{BR}\geq {\bar{\gamma }}_{P},\gamma_{CR}\geq {\bar{\gamma }}_{S}\}. \end{array}$$

Similar to the analysis for ${\mathrm{OP}}_{D}^{out}$ and ${\mathrm{OP}}_{C}^{out}$, the analytical formula for ${\mathrm{OP}}_{R}^{out}$ can be obtained by the following Lemma.

**Proposition** **3.**  At $\alpha =\alpha^{*}$, the probability at node R for retrieving the signal backscattered from node C unsuccessfully is given by

(28) OPRout=1−2mCR−1(mCR−1)!1πexp(−mBRγ¯PλBRP¯B)∑0mBR−11m!×∑k=0m(mk)(mBRγ¯PλBRP¯B)m−kG0,33,0(−mCR+12,mCR2,k|14mBRγ¯SηθλBRP¯B(mCRλCR)2).

**Proof.**  See Appendix C. □

Subsequently, the average throughput of the secondary signals computed using via the outage probability at node *C* and node *R* are given by

(29) $$\begin{array}{c} C_{C}=\left(1-P_{C}^{out}\right)\frac{{\bar{R}}_{S}}{2}, \end{array}$$

(30) $$\begin{array}{c} C_{R}=\left(1-P_{R}^{out}\right)\frac{{\bar{R}}_{S}}{2}, \end{array}$$

respectively.

With the results of $C_{D}$ in (24), $C_{C}$ and $C_{R}$ the above equations, the achievable sum-throughput can be obtained by

(31) $$\begin{array}{c} C_{\mathrm{Sum}}=C_{D}+C_{C}+C_{R}. \end{array}$$

**Remark** **2.**  *In the above equation, the throughput $C_{D}$ can be smaller than that of the traditional cooperative relaying system due to the interference from the secondary node, i.e., the AmB node C. However, the reduction in the sum-throughput can be compensated by the additional terms $C_{C}$ and $C_{R}$. The results in the next section also point out that the influence of node C to the performance of node D can be neglected by deploying a sufficient number of antennas. Consequently, the proposed network can manage to benefit from implementing AmBC.*

## 4. Numerical Results

**Figure 3:**
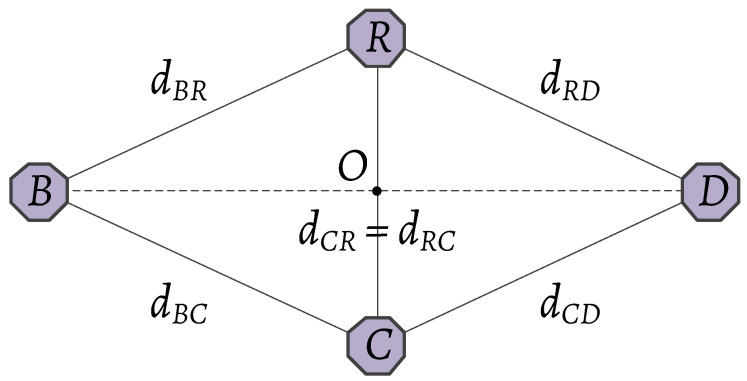
The simulation model, i.e., a parallelogram with *R*, *B*, *C* and *D* are its vertices.

In this section, simulation results are provided to evaluate the effectiveness of the proposed detectors. In the simulations, we assume that all nodes are placed in a parallelogram as Figure 3. In addition, $10^{6}$ Monte-Carlo alterations are performed to achieve reliable results. Unless there are specific modifications on the performance analysis, the default values of the different systemic parameters are set (given in Table 2). In addition, the distances $d_{BR}$ and $d_{RD}$ are determined as

$$\begin{array}{c} d_{BR}=\frac{1}{2}\sqrt{d_{RC}^{2}+d_{BD}^{2}-2d_{RC}d_{BD}\cos\left(∠ROB\right)}\;\;\mathrm{and}\;\\ d_{RD}=\frac{1}{2}\sqrt{d_{RC}^{2}+d_{BD}^{2}-2d_{RC}d_{BD}\cos\left(\pi -∠ROB\right)}, \end{array}$$

respectively.

**Figure 4:**
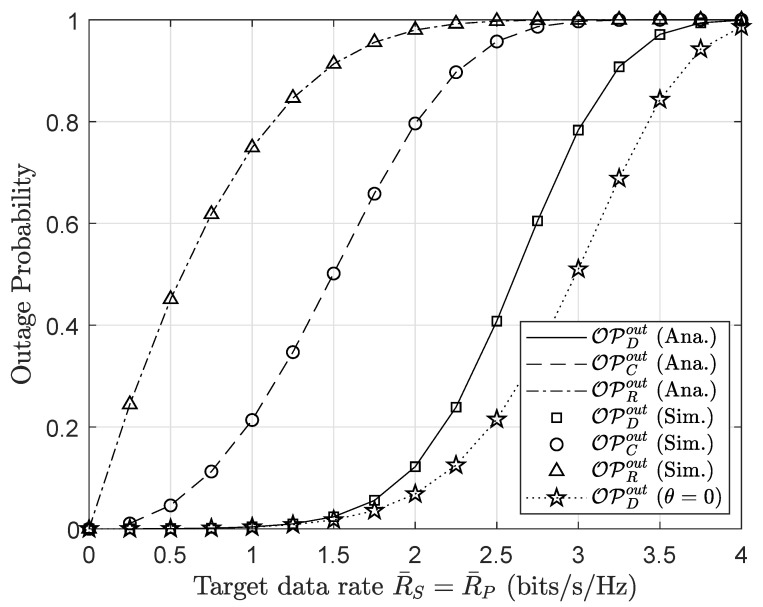
Outage performance versus the primary/secondary data rates ${\bar{R}}_{P}$, ${\bar{R}}_{S}$ (bits/s/Hz).

Figure 4 illustrates the outage performance of different nodes in such an AmBC network with respect to the range of the primary/secondary data rate. The AmBC network is beneficial with higher data rates, but a high data rate results in more severe outage performance. It is shown in Figure 4 that as the value of the transmission rate increases, the outage probabilities at the considered nodes reach one. The main reason is that outage probability is a function of data rate. At each required data rate, the outage performance of user *D* at θ=0 corresponds to the traditional cooperative relaying network where the role of AmBC is omitted. It is strongly confirmed that the analytical results match the simulation results tightly, and this observation exhibits the correctness of our analysis.

**Figure 5:**
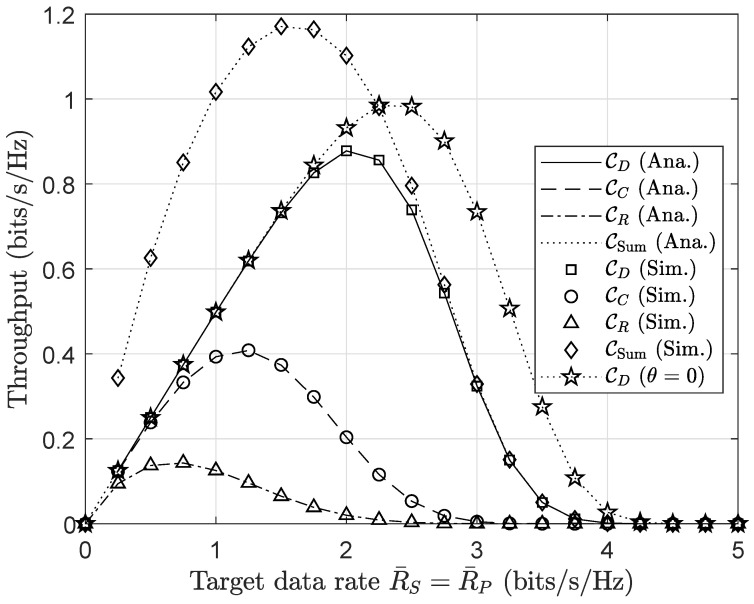
Throughput performance versus the target data rates ${\bar{R}}_{P}$, ${\bar{R}}_{S}$ (bits/s/Hz).

Four main cases are shown in Figure 5, and the highest throughput of user *D* in the case of θ=0 can be found at the target data rate of 2.5 (bits/s/Hz). It is evident that the primary communication has a leading contribution to the system’s sum-throughput, while secondary communication also provides a beneficial increment. However, the throughput starts significantly deteriorating at a higher target data rate, particularly as the data rate is beyond 3.0 (bits/s/Hz). It is the fact that the receiving nodes with a large amount of received data find difficulty to decode in the allotted time accurately. Interestingly, optimal throughput can be indicated in the numerical method by observing its fluctuation when varying transmission rates of both primary and secondary signals, i.e., ${\bar{R}}_{P}$ and ${\bar{R}}_{S}$. User *R* shows the worst performance due to a limited source of energy. This circumstance is consistent with primary evaluation in (7).

**Figure 6:**
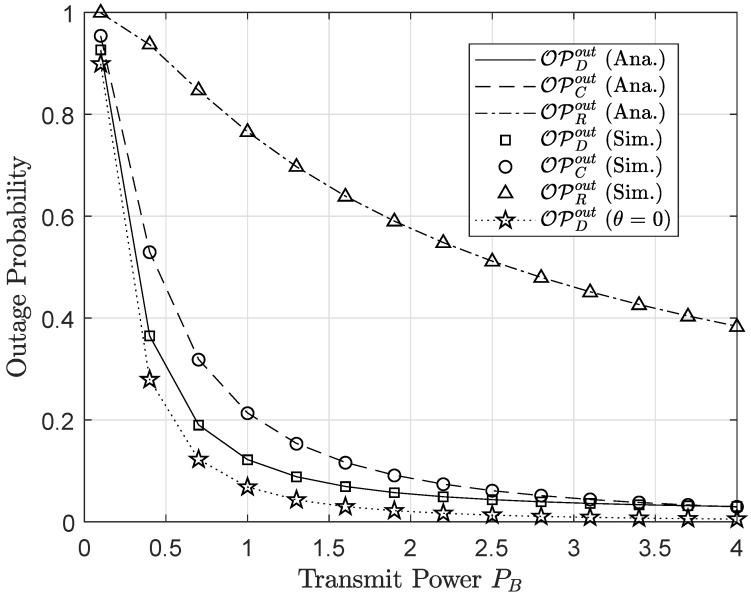
Outage performance versus transmit power ($P_{B}$) at the base station.

**Figure 7:**
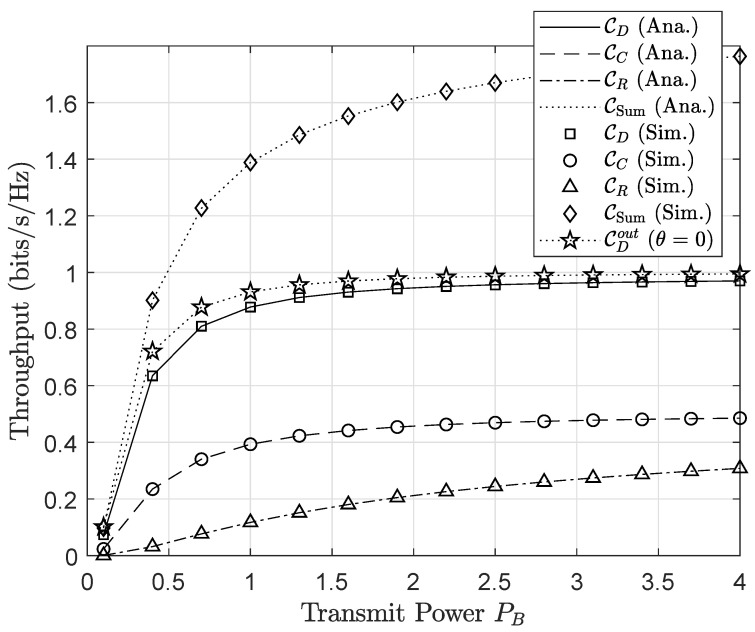
Throughput performance versus transmit power ($P_{B}$) at the base station.

Figure 6 shows that the outage performance of both primary and secondary networks would be improved by increasing the transmit power of the base station *B*. It can be explained that the higher values of the $P_{B}$ lead to the improvement of SNR/SINR at receiving nodes, which then results in reducing outage probability. More importantly, it is intuitively seen from this figure that the outage probabilities meet the saturation situation at a high $P_{B}$. It is because outage performance is limited by many parameters instead of the only value of $P_{B}$. Like previous experiments, user *D* shows the best outage performance, and this observation can be achieved thanks to increasing the MRC diversity at *D*. In a similar analysis, we plot in Figure 7 the throughput performance versus the transmit power of the base station $P_{B}$. When increasing transmit power $P_{B}$ from 0.1 W to 2 W, the throughput increases significantly, but over this point, throughput curves bend up slightly.

**Figure 8:**
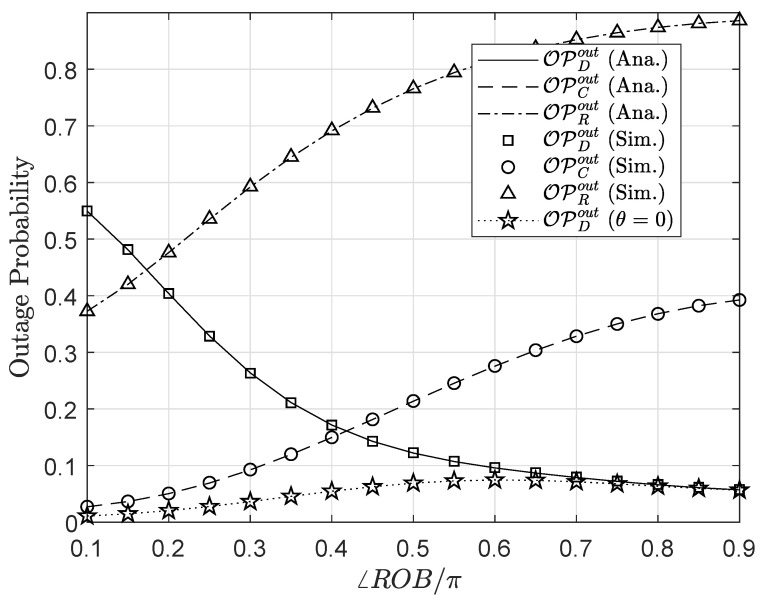
Outage performance versus the normalized angle ∠ROB.

**Figure 9:**
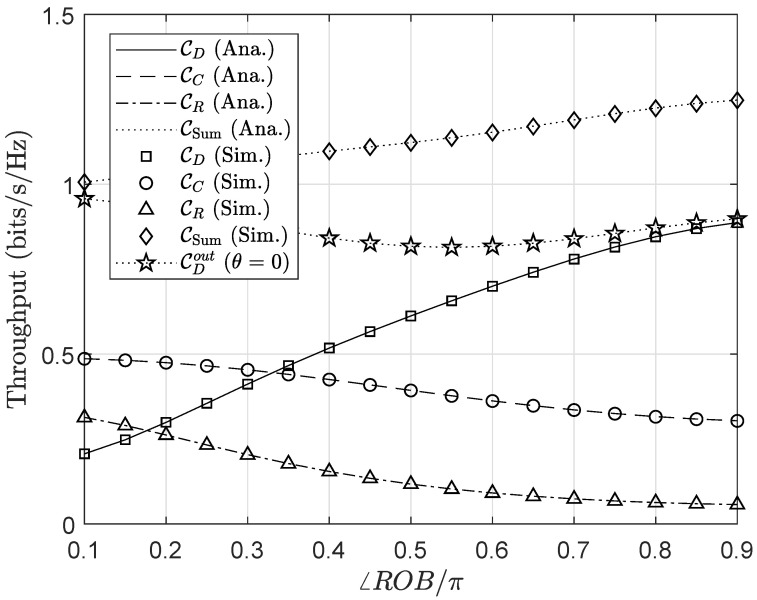
Throughput performance versus the normalized angle ∠ROB.

Figure 8 and Figure 9 exhibit the impact of the node replacement on the outage performance and throughput of the considered system. The normalized angle are varied in the range 0.1÷0.9 which represents the scenario that node *R* (node *C*) locates near the base station *B* (near node *D*) when ∠ROBπ=0.1 and then ascends further away from (nearer to) node *B* (node *D*) as ∠ROB/π surges up to 0.9. When the relay is located close to the RF source, i.e., the base station, the capability of signal receiving at *C* and *R* is drastically enhanced due to high-harvested power at node *R* offers prominent performance for the secondary network. However, in turn, it leads to a high outage and low throughput at the primary network due to excessive propagation level observed at the *R*-*D* link as well as high interference from node *C*. The scenario where the relay locates far from the node *B* can be implied analogously to the previous one.

**Figure 10:**
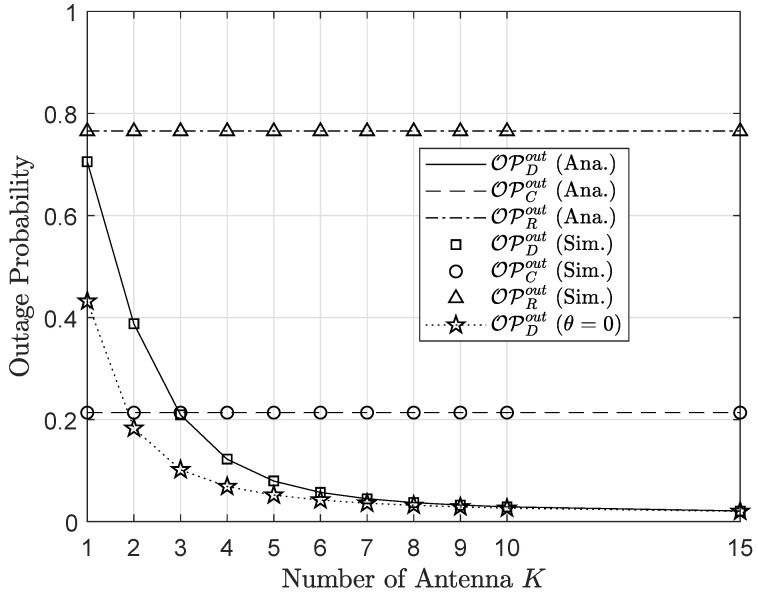
Outage probability versus the number of antenna *K*.

**Figure 11:**
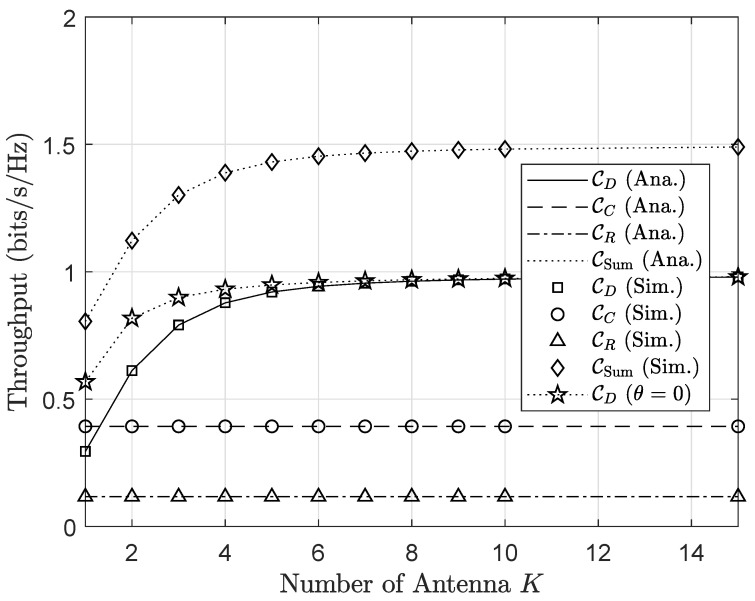
Throughput versus the number of antenna *K*.

As observation in Figure 10 and Figure 11 it can be seen the outage and throughput performance respectively. First, from Figure 10 the impact of *K* on the outage performance of three nodes *R*, *C* and *D*, where the performance improvement can be obtained by varying *K* in the range of 1÷20. However, when increasing *K* beyond 10, the outage performance changes slightly, which adapts to the common phenomenons of MRC receivers. In particular, increasing *K*, e.g., K=6,7,…, culminates in the performance of the primary network in the absence of AmBC interference. This phenomenon is one of the valuable properties of MRC receiver in mitigating co-channel interference with a drawback of complex circuitry designs. Similar trends can be seen in Figure 11 corresponding to the throughput performance.

**Figure 12:**
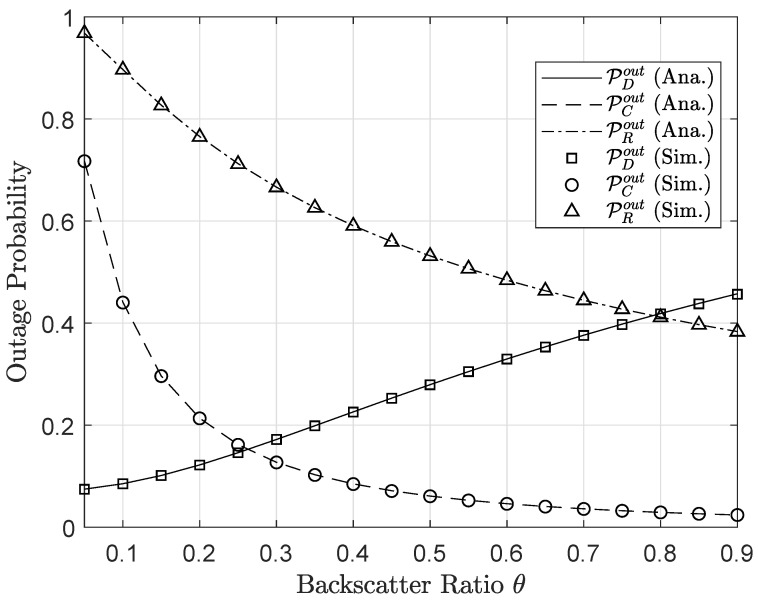
Outage probability versus different values of backscatter factor θ.

**Figure 13:**
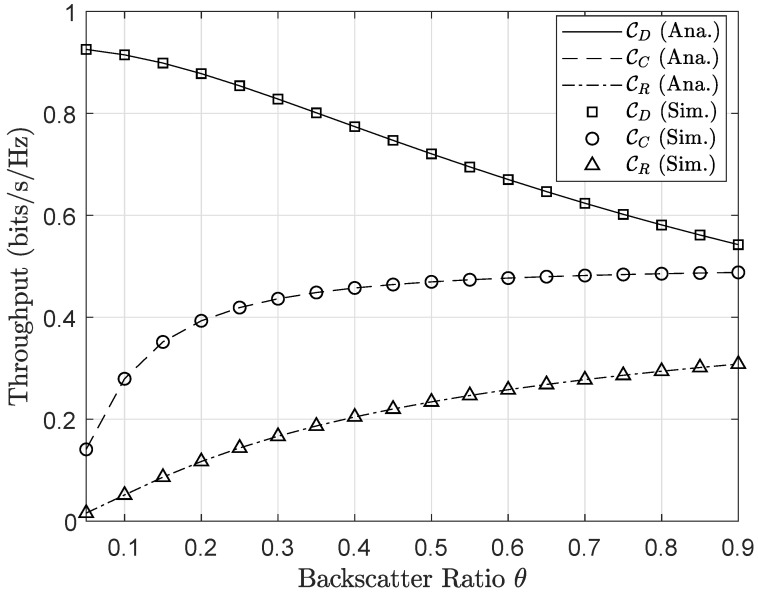
Throughput versus different values of backscatter factor θ.

In Figure 12 and Figure 13, it can be seen the significant effects of the backscattering reflection coefficient θ on the outage probability and the achievable throughput. The improvement of the secondary network’s performance is accomplished by varying values of θ. The main reason, higher values of θ leads to better SNR or SINR at both nodes *C* and *R*, and corresponding outage performance can be enhanced. In contrast, node *D* endures the impact of interference related to the backscattering reflection. Therefore, higher values of θ lead to more critical outage behavior of node *D*. It can be explained that SINR at node *D* decreases as increasing θ, and hence outage event is expected to occur at *D* more often when θ approaches to 1. The different trends of two nodes *C* and *D* provides a careful selection of θ factor to guarantee the performance of primary and secondary destinations.

## 5. Conclusions

In this paper, we have studied the system which combines AmBC, SWIPT, and cognitive radio in emerging network to obtain benefit from AmBC. We derived the exact expression of outage probability and achievable throughput at primary and secondary destinations. In section of numerical results, we show the significant impacts of different system parameters on two metrics, including outage and throughput performance. We show that the backscattering reflection coefficient can significantly change the performance of primary and secondary destinations. Furthermore, more antennas equipped at the primary destination exhibits significant improvement in system performance. Especially, the transmission power of the base station and target data rates are two main parameters affecting outage and throughput performance.

## Figures and Tables

**Table 1:** Table of abbreviations and acronyms.

**AmB**	Ambient Backscatter
**AmBC**	Ambient Backscatter Communication
**AP**	Access Point
**AWGN**	Additive White Gaussian Noise
**BD**	Backscatter Device
**CDF**	Cumulative Distribution Function
**CR**	Cognitive Radio
**CU**	Cognitive User
**DF**	Decode-and-Forward
**EH**	Energy Harvesting
**IoT**	Internet-of-Things
**IT**	Information Transmission
**MRC**	Maximal Ratio Combining
**OFDM**	Orthogonal Frequency Division Multiplexing
**PDF**	Probability Density Function
**PS**	Power-Splitting
**PU**	Primary User
**RF**	Radio Frequency
**RV**	Random Variable
**SINR**	Signal-to-Interference-plus-Noise Ratio
**SNR**	Signal-to-Noise Ratio
**SWIPT**	Simultaneous Wireless Information and Power Transfer
**TPS**	Time Power Switching
**TS**	Time Switching
**WPC**	Wireless Powered Communication

**Table 2:** Default parameters set for simulation.

Parameter	Meaning	Default Value
$P_{B}$	Transmit power of node *B*	1 W
η	EH efficiency coefficient	0.8
θ	Backscatter ratio	0.2
$\sigma^{2}$	AWGN power	$10^{-3}$ W
*K*	Number of antennas at node *D*	4
ϵ	Pathloss exponent	2
∠ROB	The angle at the vertex *O* in Figure 3	0.5 π (rad)
$d_{BD}$	Distance between node *B* and node *D*	4 (m)
$d_{RC}=d_{CR}$	Distance between node *R* and node *C*	2 (m)
$m_{RC}=m_{CR}$	Shape factor of the channel between *R* and *C*	3
$m_{BR}$	Shape factor of the channel between *B* and *R*	2
$m_{RD}$	Shape factor of the channel between *R* and *D*	2
$m_{CD}$	Shape factor of the channel between *C* and each antenna of *D*	2

## Appendix A. Proof of (17)

By using the Meijer G-function, the pdf of $X_{n}$ can be rewritten as

(A1) $$\begin{array}{cc} f_{X_{n}}\left(x_{n}\right) & =\frac{1}{\Gamma \left(m_{n}\right)}\left(\frac{m_{n}}{\mu_{n}}\right)G_{0,1}^{1,0}\left(\begin{array}{c} -\\ m_{n}-1 \end{array}|\frac{x_{n}m_{n}}{\mu_{n}}\right) \end{array}$$

(A2) $$\begin{array}{cc} \; & \overset{\left(a\right)}{=}\frac{1}{x_{n}}\frac{1}{\Gamma \left(m_{n}\right)}G_{1,0}^{0,1}\left(\begin{array}{c} 1-m_{n}\\ - \end{array}|\frac{\mu_{n}}{x_{n}m_{n}}\right), \end{array}$$

where (a) is the result of using [35] (Equations (9.31.2) and (9.31.5)).

Accordingly, the PDF of *Z* conditioned on $X_{n}$ (n≥2) can be expressed as

(A3) $$\begin{array}{cc} f_{Z}\left(z|X_{2},\ldots ,X_{N}\right)= & \frac{1}{z}\frac{1}{\Gamma \left(m_{1}\right)}G_{1,0}^{0,1}\left(\begin{array}{c} 1-m_{1}\\ - \end{array}|\frac{\mu_{1}X_{2}\cdots X_{N}}{zm_{1}}\right). \end{array}$$

By taking the expectation of $f_{Z}\left(z|X_{2},\ldots ,X_{N}\right)$ over $X_{2}$ using the PDF $f_{X_{2}}\left(x_{2}\right)$ in (A1), i.e., $E_{X_{2}}\left[f_{Z}\left(z|X_{2},\ldots ,X_{N}\right)\right]$, we then obtain the PDF of *Z* conditioned on $X_{n}$ (n≥3) as

$$\begin{array}{cc} f_{Z}\left(z|X_{3},\ldots ,X_{N}\right)= & \frac{1}{z}\frac{1}{\Gamma \left(m_{1}\right)\Gamma \left(m_{2}\right)}\frac{m_{2}}{\mu_{2}} \end{array}$$

(A4) $$\begin{array}{cc} \; & \times \int_{0}^{\infty }G_{0,1}^{1,0}\left(\begin{array}{c} -\\ m_{2}-1 \end{array}|\frac{x_{2}m_{2}}{\mu_{2}}\right)G_{1,0}^{0,1}\left(\begin{array}{c} 1-m_{1}\\ - \end{array}|x_{2}\frac{\mu_{1}}{zm_{1}}[\prod_{n=3}^{N}X_{n}]\right)dx_{2} \end{array}$$

(A5) $$\begin{array}{cc} = & \frac{1}{z}\frac{1}{\Gamma \left(m_{1}\right)\Gamma \left(m_{2}\right)}G_{2,0}^{0,2}\left(\begin{array}{c} 1-m_{2},1-m_{1}\\ - \end{array}|\frac{1}{z}\frac{\mu_{1}}{m_{1}}\frac{\mu_{2}}{m_{2}}[\prod_{n=3}^{N}X_{n}]\right). \end{array}$$

By taking the expectation of the above equation over $X_{n}$ (n≥3) repeatedly, the marginal PDF of *Z* can then be obtained as

(A6) $$\begin{array}{cc} f_{Z}\left(z\right)= & \frac{1}{z}\left(\prod_{n=1}^{N}\frac{1}{\Gamma \left(m_{n}\right)}\right)G_{N,0}^{0,N}\left(\begin{array}{c} 1-m_{N},\ldots ,1-m_{2},1-m_{1}\\ - \end{array}|\frac{1}{z}\prod_{n=1}^{N}\frac{\mu_{n}}{m_{n}}\right) \end{array}$$

(A7) $$\begin{array}{cc} = & \frac{1}{z}\left(\prod_{n=1}^{N}\frac{1}{\Gamma \left(m_{n}\right)}\right)G_{0,N}^{N,0}\left(\begin{array}{c} -\\ m_{N},\ldots ,m_{2},m_{1} \end{array}|z\prod_{n=1}^{N}\frac{m_{n}}{\mu_{n}}\right), \end{array}$$

in which the last equality is the result of adopting [35] (Equation (9.31.2)). Moreover, with the aid of [35] (Equation (9.31.5)), we then obtain (17). This is the end the the proof.

## Appendix B. Proof of Proposition 1

We first rewrite the probability $P_{D}^{out}$ in (20) as $P_{D}^{out}=1-\mathrm{Pr}\left\{\gamma_{BR}\geq {\bar{\gamma }}_{P},\gamma_{RD}^{MRC}\geq {\bar{\gamma }}_{P}\right\}$ and then adopt $\gamma_{BR}$ and $\gamma_{RD}^{MRC}$ in (4) and (11), respectively, thus

(A8) $$\begin{array}{c} {\mathrm{OP}}_{D}^{out}=1-\mathrm{Pr}\{\eta \left({\bar{P}}_{B}X_{BR}-{\bar{\gamma }}_{P}\right)\geq 0,\frac{\eta \left({\bar{P}}_{B}X_{BR}-{\bar{\gamma }}_{P}\right)X_{RD}}{\eta \left({\bar{P}}_{B}X_{BR}-{\bar{\gamma }}_{P}\right)X_{RC}X_{CD}+1}\geq {\bar{\gamma }}_{P}\}. \end{array}$$

Let $W=\eta \left({\bar{P}}_{B}X_{BR}-{\bar{\gamma }}_{P}\right){𝟙}_{Z}$, in which ${𝟙}_{Z}=1$ if $X_{BR}>{\bar{\gamma }}_{P}/{\bar{P}}_{B}$, otherwise, ${𝟙}_{Z}=0$. In addition, the CDF of the random variable *W* is given by

(A9) $$\begin{array}{cc} F_{W}\left(w\right)= & F_{X_{BR}}\left(\frac{w+\eta {\bar{\gamma }}_{P}}{\eta {\bar{P}}_{B}}\right),\;w>0, \end{array}$$

(A10) $$\begin{array}{cc} = & 1-\exp\left(-\frac{m_{BR}}{\lambda_{BR}}\frac{w+\eta {\bar{\gamma }}_{P}}{\eta {\bar{P}}_{B}}\right)\sum_{m=0}^{m_{BR}-1}\frac{1}{m!}{\left(\frac{m_{BR}}{\lambda_{BR}}\frac{w+\eta {\bar{\gamma }}_{P}}{\eta {\bar{P}}_{B}}\right)}^{m},\;w>0, \end{array}$$

thus the PDF *W* is obtained by taking the first derivative of $F_{W}\left(w\right)$ which is given by

(A11) $$\begin{array}{cc} f_{W}\left(w\right)= & \frac{\partial }{\partial w}F_{W}\left(w\right)=\frac{1}{\eta {\bar{P}}_{B}}f_{X_{BR}}\left(\frac{w+\eta {\bar{\gamma }}_{P}}{\eta {\bar{P}}_{B}}\right),\;w>0, \end{array}$$

(A12) $$\begin{array}{cc} = & \frac{{\left(w+\eta {\bar{\gamma }}_{P}\right)}^{m_{BR}-1}}{\left(m_{BR}-1\right)!}{\left(\frac{m_{BR}}{\eta {\bar{P}}_{B}\lambda_{BR}}\right)}^{m_{BR}}\exp\left(-\frac{m_{BR}}{\eta {\bar{P}}_{B}\lambda_{BR}}\left(w+\eta {\bar{\gamma }}_{P}\right)\right),\;w>0, \end{array}$$

Subsequently, the probability ${\mathrm{OP}}_{D}^{out}$ in (A8) can be rewritten as

(A13) $$\begin{array}{cc} {\mathrm{OP}}_{D}^{out}= & 1-\mathrm{Pr}\{\frac{WX_{RD}}{WX_{RC}X_{CD}+1}\geq {\bar{\gamma }}_{P}\} \end{array}$$

(A14) $$\begin{array}{cc} = & 1-\int_{0}^{\infty }\int_{0}^{\infty }\mathrm{Pr}\{X_{RD}\geq {\bar{\gamma }}_{P}\left(y+\frac{1}{w}\right)\}f_{X_{RC}X_{CD}}\left(y\right)f_{W}\left(w\right)dydw\\ = & 1-\sum_{m=0}^{Km_{RD}-1}\frac{1}{m!}{\left(\frac{m_{RD}{\bar{\gamma }}_{P}}{\lambda_{RD}}\right)}^{m}\int_{0}^{\infty }\int_{0}^{\infty }{\left(y+\frac{1}{w}\right)}^{m} \end{array}$$

(A15) $$\begin{array}{cc} \; & \;\times \exp\{-\frac{m_{RD}{\bar{\gamma }}_{P}}{\lambda_{RD}}\left(y+\frac{1}{w}\right)\}f_{X_{RC}X_{CD}}\left(y\right)f_{W}\left(w\right)dydw. \end{array}$$

With the help of the binomial theorem, the calculation for the last integral can be performed as

(A16) OPDout=1−∑m=0KmRD−11m!(mRDγ¯PλRD)m∑m1+m2=m(mm1,m2)×∫0∞ym1exp(−mRDγ¯PλRDy)fXRCXcd(y)dy︸P1(m1)∫0∞1wm2exp(−mRDγ¯PλRD1w)fW(w)dw︸P2(m2).

Let denote the first and the second integral by $P_{1}$ and $P_{2}$, respectively. Using the pdf of $X_{RC}X_{CD}$ in (19), the first integral part ($P_{1}$) can be derived as

(A17) $$\begin{array}{cc} P_{1}\left(m_{1}\right)= & \frac{1}{\left(m_{RC}-1\right)!\left(m_{CD}-1\right)!}\frac{A}{2}{\left(\frac{A}{4}\right)}^{\frac{m_{RC}+m_{CD}}{2}-1}\\ \; & \times \int_{0}^{\infty }y^{\frac{m_{RC}+m_{CD}}{2}+m_{1}-1}\exp\left(-\frac{m_{RD}{\bar{\gamma }}_{P}}{\lambda_{RD}}y\right)K_{m_{RC}-m_{CD}}\left(\sqrt{Ay}\right)dy. \end{array}$$

In addition, the integral $P_{2}$, after substituting $f_{W}\left(w\right)$ in (A12) into $P_{2}\left(m_{2}\right)$, is given by

(A18) $$\begin{array}{cc} P_{2}\left(m_{2}\right)= & \frac{1}{\left(m_{BR}-1\right)!}{\left(\frac{m_{BR}}{\eta {\bar{P}}_{B}\lambda_{BR}}\right)}^{m_{BR}}\exp\left(-\frac{m_{BR}{\bar{\gamma }}_{P}}{{\bar{P}}_{B}\lambda_{BR}}\right)\\ \; & \times \int_{0}^{\infty }\frac{{\left(w+\eta {\bar{\gamma }}_{P}\right)}^{m_{BR}-1}}{w^{m_{2}}}\exp\left(-\frac{m_{RD}{\bar{\gamma }}_{P}}{\lambda_{RD}}\frac{1}{w}-\frac{m_{BR}}{\eta {\bar{P}}_{B}\lambda_{BR}}w\right)dw, \end{array}$$

## Appendix C. Proof of Proposition 3

At $\alpha =\alpha^{*}$ in (7), the probability ${\mathrm{OP}}_{R}^{out}$ can be given by

(A19) $$\begin{array}{c} {\mathrm{OP}}_{R}^{out}=1-\mathrm{Pr}\left\{\left({\bar{P}}_{B}X_{BR}-{\bar{\gamma }}_{P}\right)\eta >0,X_{CR}^{2}\left({\bar{P}}_{B}X_{BR}-{\bar{\gamma }}_{P}\right)\eta \theta \geq {\bar{\gamma }}_{S}\right\}=1-\mathrm{Pr}\left\{X_{CR}^{2}W\theta \geq {\bar{\gamma }}_{S}\right\}, \end{array}$$

where the random variable *W* as well as its PDF/CDF are evaluated in the Appendix B.

Subsequently, we can rewrite ${\mathrm{OP}}_{R}^{out}$ as

(A20) $$\begin{array}{cc} {\mathrm{OP}}_{R}^{out}= & 1-\int_{0}^{\infty }\exp\left(-\frac{m_{BR}}{\lambda_{BR}}\frac{\frac{{\bar{\gamma }}_{S}}{\theta x_{CR}^{2}}+\eta {\bar{\gamma }}_{P}}{\eta {\bar{P}}_{B}}\right)\sum_{m=0}^{m_{BR}-1}\frac{1}{m!}{\left(\frac{m_{BR}}{\lambda_{BR}}\frac{\frac{{\bar{\gamma }}_{S}}{\theta x_{CR}^{2}}+\eta {\bar{\gamma }}_{P}}{\eta {\bar{P}}_{B}}\right)}^{m}f_{X_{CR}}\left(x\right)dx. \end{array}$$

Substituting the PDF $f_{X_{CR}}\left(x\right)$ in (1) into the above integral, the outage probability ${\mathrm{OP}}_{R}^{out}$ can then be expressed as

(A21) $$\begin{array}{cc} {\mathrm{OP}}_{R}^{out}= & 1-\frac{1}{\left(m_{CR}-1\right)!}\exp\left(-\frac{m_{BR}{\bar{\gamma }}_{P}}{{\bar{P}}_{B}\lambda_{BR}}\right){\left(\frac{m_{CR}}{\lambda_{CR}}\right)}^{m_{CR}}\sum_{m=0}^{m_{BR}-1}\frac{1}{m!}{\left(\frac{m_{BR}{\bar{\gamma }}_{P}}{{\bar{P}}_{B}\lambda_{BR}}\right)}^{m}\\ \; & \times \int_{0}^{\infty }x^{m_{CR}-1}{\left(1+\frac{{\bar{\gamma }}_{S}}{\eta \theta {\bar{\gamma }}_{P}}\frac{1}{x^{2}}\right)}^{m}\exp\left(-\frac{m_{BR}{\bar{\gamma }}_{P}}{\eta \theta {\bar{P}}_{B}\lambda_{BR}}\frac{1}{x^{2}}-\frac{m_{CR}}{\lambda_{CR}}x\right)dx. \end{array}$$

The above integral, denoted by I, can be expanded as

(A22) I=∑k=0m(mk)(γ¯Sηθγ¯P)k∫0∞xmCR−2k−1exp(−mBRγ¯PηθP¯BλBR1x2−mCRλCRx)dx=∑k=0m(mk)(γ¯Sηθγ¯P)k(mBRγ¯SηθλBRP¯B)mCR−12−k

(A23) $$\begin{array}{cc} \; & \;\times \int_{0}^{\infty }\exp\left(-\frac{m_{CR}}{\lambda_{CR}}x\right)G_{1,0}^{0,1}\left(\begin{array}{c} \frac{m_{CR}+1}{2}-k\\ - \end{array}|\frac{\eta \theta \lambda_{BR}{\bar{P}}_{B}}{m_{BR}{\bar{\gamma }}_{s}}x^{2}\right)dx, \end{array}$$

in which the last equation is derived with the help of [35] (Equation (7.813.2)). Thus, with the above results, we then obtain the desired analytical form of ${\mathrm{OP}}_{R}^{out}$ in (28).
